# Unlocking the Potential of Oleanolic Acid: Integrating Pharmacological Insights and Advancements in Delivery Systems

**DOI:** 10.3390/pharmaceutics16060692

**Published:** 2024-05-21

**Authors:** Muhammad Wasim, Maria Camilla Bergonzi

**Affiliations:** Department of Chemistry, University of Florence, Via U. Schiff 6, 50019 Sesto Fiorentino, Italy; muhammad.wasim@unifi.it

**Keywords:** oleanolic acid, solubility, bioavailability, drug delivery systems

## Abstract

The growing interest in oleanolic acid (OA) as a triterpenoid with remarkable health benefits prompts an emphasis on its efficient use in pharmaceutical research. OA exhibits a range of pharmacological effects, including antidiabetic, anti-inflammatory, immune-enhancing, gastroprotective, hepatoprotective, antitumor, and antiviral properties. While OA demonstrates diverse pharmacological effects, optimizing its therapeutic potential requires overcoming significant challenges. In the field of pharmaceutical research, the exploration of efficient drug delivery systems is essential to maximizing the therapeutic potential of bioactive compounds. Efficiently delivering OA faces challenges, such as poor aqueous solubility and restricted bioavailability, and to unlock its full therapeutic efficacy, novel formulation strategies are imperative. This discussion thoroughly investigates different approaches and advancements in OA drug delivery systems with the aim of enhancing the biopharmaceutical features and overall efficacy in diverse therapeutic contexts.

## 1. Introduction

Oleanolic acid (OA), a triterpenoid sourced from nature, is emerging as a promising compound for its potential health benefits. OA has diverse pharmacological properties, including antidiabetic, anti-inflammatory, immune-boosting, gastroprotective, hepatoprotective, antitumor, and antiviral activities [1,2]. In the last decade, a growing interest and advancement in scientific understanding of OA has been observed [3]. These reports cover the following topics: the pharmacological investigation of the plant-derived triterpene’s invaluable effects [4,5,6,7,8,9,10], bioavailability and toxicological studies [10,11,12,13,14], and the clinical application of OA in the prevention of various ailments, primarily chronic diseases, with inflammation and oxidative stress as the main mechanisms [15,16,17,18]. According to the Biopharmaceutical Classification System (BCS), OA is a BCS IV drug, with limited aqueous solubility and permeability being the main reasons for its restricted application in health [19]. Despite its innumerable biological effects, OA has poor aqueous solubility (1.75 μg/mL) with low bioavailability, similar to many other natural substances. OA’s oral bioavailability is only 0.7% for oral doses of 25 and 50 mg/kg in rats, due to its poor solubility and dissolution rate [20]. Therefore, its use in the pharmaceutical field is rather limited [3]. Many approaches have been attempted to address these issues. This review presents a current overview of the key developments in the OA delivery systems and the recent findings and research progress made in the formulation of OA to enhance the biopharmaceutical features and overall efficacy in diverse therapeutic contexts. Delivering therapeutic compounds effectively is a challenge. Traditional formulations often lack precision and can cause side effects. Controlled drug delivery systems offer a solution by transporting drugs directly to target sites, minimizing side effects, and reducing required dosages.

There are numerous examples of application of the drug delivery systems (DDS) in this field related to isolated products from vegetable origin or purified extracts that contain different classes of active ingredients [21].

In conducting this review, a comprehensive literature search was performed across multiple databases, including PubMed, Scopus, and the Web of Science. Keywords such as biological and pharmacological activities of oleanolic acid, molecular mechanisms of oleanolic acid, oleanolic acid delivery, oleanolic acid formulation, and specific drug delivery systems for oleanolic acid, such as liposomes and nanoparticles, were used to identify relevant studies. Inclusion and exclusion criteria were applied based on study relevance to ensure the selection of pertinent literature for analysis.

### 1.1. Chemical Structure and Properties of OA

**Figure 1:**
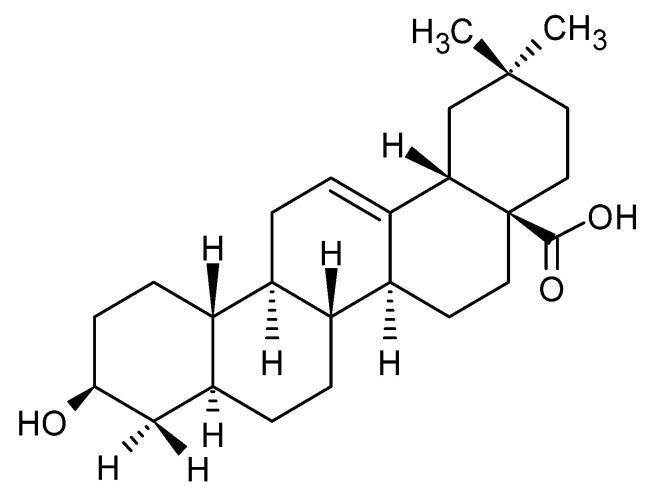
Chemical structure of oleanolic acid.

OA has three distinct functional groups: a β-hydroxy at position 3, a carboxyl at position 28, and a double bond between positions 12 and 13, as shown in Figure 1. This compound occurs naturally in a free acid form and serves as the aglycone for various triterpenoid saponins bound to sugar molecules. It appears as a colorless, tasteless, and odorless solid, demonstrating minimal solubility in water [22]. OA has a molecular weight of 456.7 g/mol [3]. It is less hygroscopic because of its hydrophobic nature [23]. The biogenesis of OA and steroids follows a similar pathway, which includes the cyclization of squalene, a common precursor for both compounds. OA primarily features the oleanane skeleton as its core structure [24].

### 1.2. Natural Sources of OA and Its Extraction

OA is a naturally occurring pentacyclic triterpenoid isolated from several food and medicinal plants [25]. OA is named after the *Olea europaea* L. plant, which is the primary source of commercial OA products [26]. OA is found in various plant families, such as Araliaceae, Asteraceae, Ericaceae, Lamiaceae, Myrtaceae, Oleaceae, Rosaceae, Rubiaceae, Saxifragaceae, and Verbenaceae [25,27,28]. Different parts of these plants may contain varying amounts of OA. For instance, *Olea europaea* leaves have been found to have more OA (31 mg/g dry weight) compared to the bark (up to 9.8 mg/g dry weight) [25,29].

Compounds such as OA and related triterpenes have low solubility in water and other common organic solvents. They also tend to become saturated with co-extracted metabolites [30,31]. To improve extraction, medium-polar solvents should be chosen with a Hildebrand solubility parameter (δ) in the range of 10 to 12. The n-butanol solvent (δ = 10.4) is the best performer due to its compatibility with OA’s solubility parameter, which is reported as δ 10.2. Solvents such as ethyl ether, chloroform, methanol, and ethanol also yield high results, while nonpolar solvents such as toluene and cyclohexane are less effective [31,32]. Ethanol and methanol have matching polarities with OA, leading to high extraction yields. Ethanol is the preferred choice in the food, cosmetic, and pharmaceutical industries due to its lower toxicity. Aqueous ethanol (EtOH:H20 70:30 *v*/*v*) is found to be a better solvent than absolute ethanol, as indicated by different studies [33,34].

## 2. Pharmacological Activities of OA

**Figure 2:**
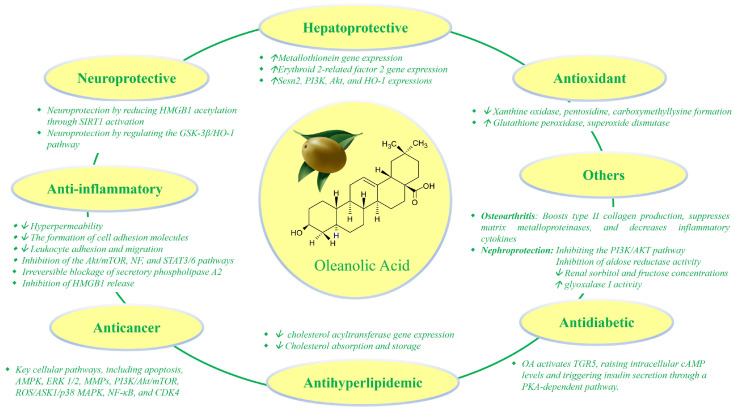
OA pharmacological activities.

OA has been the subject of extensive research due to its diverse pharmacological activities (Figure 2). Below are some of the pharmacological activities of OA.

### 2.1. Anti-Inflammatory Properties

OA is recognized for its anti-inflammatory behavior by preventing hyperpermeability, the formation of cell adhesion molecules, and leukocyte adhesion and migration, indicating its potential as a treatment for vascular inflammatory diseases [35]. It has been found that the main anti-inflammatory property of OA is mediated through the inhibition of the Akt/mTOR, NF, and STAT3/6 pathways [36]. Moreover, OA forms a complex with secretory phospholipase A2, a crucial enzyme in inflammatory responses, which causes irreversible blockage [18]. In a study, OA protected against intestinal damage caused by dextran sulfate sodium (DSS) in mice. It countered increased TGR5 and BAX expression and decreased Bcl2 expression induced by DSS [37]. The high-mobility group box 1 (HMGB1) protein, known for its role in inflammation, exacerbates pro-inflammatory responses and correlates with a poor prognosis in severe inflammation with high mortality. OA effectively inhibits HMGB1 release and subsequent pro-inflammatory effects on human endothelial cells. These findings highlight OA’s potential therapeutic value in inflammatory diseases by targeting the HMGB1 pathway [38]. Studies explored OA’s influence on eicosanoid production in human coronary smooth-muscle cells. OA stimulated the release of prostaglandin I2 (PGI2) via upregulation of Cox-2. This effect was blocked by a Cox-2 inhibitor. OA also activated p38 and p42/44 MAPK pathways, contributing to Cox-2 upregulation and PGI2 release. These findings suggest OA helps maintain vascular health by promoting PGI2 release through Cox-2 activation [39]. The inhibitory effects of OA on allergic inflammation using human mast cells and a mouse model of anaphylactic shock have also been investigated. OA suppressed pro-inflammatory cytokines and histamine release by inhibiting the Akt, p38 MAPK, NF-κB, and STAT1 signaling pathways. These findings suggest that OA could serve as a therapeutic option for allergic disorders, including anaphylaxis [40].

### 2.2. Antioxidant Effects

OA does not just act as a free radical-scavenger through direct chemical reactions—it also serves as a biological molecule, potentially boosting antioxidant defenses [41]. OA also demonstrated significant superoxide anion scavenging activity and dose-dependent inhibition of xanthine oxidase, pentosidine, and carboxymethyl lysine formation [42]. In a study on Dahl salt-sensitive rats, OA exhibited potent antioxidant activity, increasing glutathione peroxidase by 12% and superoxide dismutase by 12%. This suggests OA’s potential in combating oxidative stress, a crucial factor in hypertension and related complications [43]. OA also showed an ability for enhancing protection against oxidative stress in macrophages [44].

### 2.3. Anticancer Potential

OA has gained significant attention owing to its remarkable antitumor effects and pharmacological safety [45]. Research shows that OA has a significant effect on treating different tumor cell lines, including human breast cancer cells, such as MCF-7 and MCF-7/ADR, the astrocytoma cell line 1321N1, hepatocellular carcinoma, colorectal cancer cells (HCT-116), and various others [46]. Moreover, OA showed antimutagenic effects in Balb/c mice, as evidenced by the micronucleus test conducted on peripheral blood and bone marrow [47]. In cancer, the inflammation is pivotal for development and progression. NF-κB, a key factor in inflammation, is often overexpressed in cancer cells, suppressing tumor cell apoptosis and sustaining a pro-cancer inflammatory environment. OA shows a promising effect on countering inflammation and cancer, potentially by targeting NF-κB, though its precise mechanism is still unknown [48]. The potential of OA in cancer treatment is highlighted by its impact on key cellular pathways, including apoptosis, AMPK, ERK 1/2, MMPs, PI3K/Akt/mTOR, ROS/ASK1/p38 MAPK, NF-κB, and CDK4 [49].

### 2.4. Hepatoprotective Activity

OA has been employed in China for decades as an over-the-counter hepatoprotective drug [50]. OA is renowned for its ability to provide hepatoprotection in cases of acute, chemically induced liver injury, chronic liver fibrosis, and cirrhosis [51]. A research study revealed that hepatoprotective doses of OA in rats and mice elicited a consistent pattern of gene expression changes, especially significant increases in the expression of genes related to metallothionein and erythroid 2-related factor 2 (Nrf2). These findings offer new insights into the broad hepatoprotective mechanisms of OA [52]. Another study explored OA’s protective effects against carbon-tetrachloride-induced liver damage in mice: OA pretreatment significantly reduced liver enzyme activity and lipid peroxidation while preserving glutathione levels. It also inhibited the activity and expression of cytochrome P450 2E1, the enzyme responsible for carbon tetrachloride activation [53]. Furthermore, the combination of OA and ursolic acid (UA) prevents the steatosis induced by anti-TB drugs [54]. The precise hepatoprotective mechanism of OA remains uncertain [55], but Hao et al. explored its effect against hepatic ischemia-reperfusion injury through the HO-1/Sesn2 pathway, which involves the upregulation of Sesn2, PI3K, Akt, and HO-1 expressions [56].

### 2.5. Neuroprotective Activity

Neuroprotective potential is evident across various studies on OA. In cell studies, OA suppressed the release of IL-1β, IL-6, TNF-α, and NO, suggesting its neuroprotective role against oxidative stress and inflammation linked to Alzheimer’s disease [57]. In rat models, OA administered before inducing cortical hypoxia demonstrated neuroprotective effects by suppressing harmful glial activities and promoting neuronal survival [58]. Additionally, OA showed potential in protecting against stroke-induced injury in mice, providing both short-term antioxidant benefits and long-term neuroprotection [59]. Moreover, in subarachnoid hemorrhage studies, OA exhibited neuroprotection by reducing HMGB1 acetylation through SIRT1 activation, demonstrating anti-inflammatory effects and decreasing TLR4 levels [60]. OA also reduced cytotoxicity and ROS levels in vitro, decreased cerebral infarction, and improved neurological scores in rat models of ischemia. It also increased cell survival and reduced apoptosis in the infarct area while regulating the GSK-3β/HO-1 pathway [61]. The molecular dynamic simulations and docking, along with biomimetic tests, explored OA’s potential as an AChE inhibitor. In vitro tests with SH-SY5Y cells indicated reduced cell viability at higher concentrations, with an IC50 value of 714.32 ± 32.40 µg/mL. In vivo tests on zebrafish showed no significant differences in mortality rate or morphology between the control and experimental groups. CH-π interactions between OA and aromatic side chains were observed, revealing insights into its inhibitory mechanism against AChE [62].

### 2.6. Other Therapeutic Effects

OA offers diverse therapeutic benefits, including managing diabetes and supporting heart and kidney functions. In an investigation, OA demonstrated prominent effects in reducing blood glucose levels and promoting weight loss in diabetic animals induced by streptozotocin. Additionally, in an insulin-resistant model, OA could enhance insulin signal transduction. These results significantly contributed to advancing our comprehension of the pharmacological effects of OA in diabetes mellitus [63]. A study investigated OA effects on hepatic insulin resistance in obese diabetic mice. OA administration for two weeks improved glucose tolerance, insulin signaling, and reduced liver fat accumulation. It also lowered blood glucose, triglycerides, cholesterol levels, and inflammatory markers. These results suggest OA’s potential for treating insulin resistance and related metabolic disorders [64]. By acting as a TGR5 agonist, OA decreases serum glucose and insulin levels in mice on a high-fat diet, thereby enhancing glucose tolerance [65]. TGR5, found in pancreatic tissue, influences beta cell function. OA activates TGR5, increasing intracellular cAMP levels and stimulating insulin secretion via a PKA-dependent pathway. OA affects KATP and Ca^2+^ currents, enhancing stimulus-secretion coupling in beta cells [66]. The therapeutic effects of OA on hyperlipidemia induced by a high-cholesterol diet in rats were also explored. OA supplementation for 15 days reduced serum cholesterol levels, suppressed Acyl-CoA cholesterol acyltransferase gene expression, and decreased hepatic lipid accumulation. These findings suggest that OA has potential therapeutic benefits against diet-induced hyperlipidemia by inhibiting cholesterol absorption and storage [67]. OA has demonstrated clear biological activity, including antibacterial, antiviral, and antiprotozoal effects [28]. Moreover, OA has been shown to decrease and mitigate the severity and progression of experimental autoimmune encephalomyelitis. Hence, using OA for therapeutic purposes carries considerable clinical significance in the treatment of human multiple sclerosis [68].

A study examined the effects of OA on aldose-reductase and glycation products in diabetic mice. OA intake reduced glucose levels, increased insulin, and improved kidney function. It also lowered AR activity, decreased renal sorbitol and fructose concentrations, and enhanced glyoxalase I activity. These findings suggest that OA may help prevent or alleviate glycation-related kidney diseases [69].

Obesity-related inflammation, driven by adipose tissue macrophages, is a key factor in metabolic disorders. OA showed a significant antidiabetic and anti-inflammatory effect. In obese mice, OA improved insulin resistance and reduced adipose tissue inflammation. It also lowered pro-inflammatory markers in both mice and cultured macrophages (RAW 264.7). OA targeted mitochondrial function and macrophage activation by inhibiting MAPK signaling and NLRP3 inflammasome activation [70].

The cardioprotective effects of OA against doxorubicin (Dox)-induced cardiotoxicity in rats were also probed. Dox administration caused cardiac damage, evidenced by altered blood pressure, left ventricular function, and biochemical markers. OA significantly protected against Dox-induced toxicity, indicating OA’s potential as a protective therapy alongside Dox in cancer treatment [71].

In a study on mice with testosterone-induced androgenetic alopecia, the effects of different concentrations of OA on hair growth and related mechanisms were examined. Mice treated with 1% or 0.5% OA showed significantly improved hair growth, along with increased levels of key factors, such as vascular endothelial growth factor and fibroblast growth factor receptor. Immunofluorescence staining revealed significant expression of β-catenin, suggesting that OA stimulates hair growth via the Wnt/β-catenin pathway while also reducing levels of factors inhibiting hair growth [72].

Another study explored how OA resists extracellular matrix degradation and its protective effects in osteoarthritis. OA boosts type II collagen production, suppresses matrix metalloproteinases, and decreases inflammatory cytokines and cartilage markers, thereby impeding cartilage degeneration. OA also hinders the Wnt/β-catenin pathway, triggers the Hippo/YAP pathway, and prevents ECM degradation by blocking β-catenin and YAP nuclear translocation [73]. Recently, interest has grown in the impact of bile acids and their receptor, TGR5, on muscle function and metabolic health. Increasing slow muscle fiber content is seen as beneficial for metabolic health. OA-activating TGR5 was studied for its effect on muscle fiber types and associated mechanisms. Mice were supplemented with 0, 50, or 100 mg/kg of OA, and C2C12 cells were treated with various OA concentrations. OA promoted conversion from fast to slow muscle fibers by activating the TGR5-mediated calcineurin/nuclear factor of activated T cells cytoplasmic 1 (NFATc1) pathway. Inhibiting TGR5 and calcineurin reversed OA’s effects on muscle fiber transformation [74]. The effects of OA on skeletal muscle production and proliferation of C2C12 cells were investigated. It was found that OA significantly boosts skeletal muscle mass, improves glucose intolerance and insulin resistance, and inhibits dexamethasone-induced muscle atrophy by regulating the PI3K/Akt pathway. Moreover, OA reduces the expression of atrophy-related genes in the skeletal muscle of obese mice and enhances PI3K/Akt activation, promoting protein synthesis and alleviating muscle atrophy [75]. A study investigated OA’s therapeutic effects on renal ischemia reperfusion injury (RIRI) and its mechanism. Rats with RIRI were treated with OA alone, LY294002 (a PI3K inhibitor) alone, or their combination. Results showed improved kidney symptoms and decreased Cr levels in all treatment groups. Expression levels of PI3K/Akt pathway components were significantly reduced in treated groups compared to the RIRI model group. This suggests that OA may alleviate RIRI symptoms by inhibiting the PI3K/Akt pathway [76].

In summary, OA exhibits a wide range of therapeutic benefits, including antidiabetic, anti-inflammatory, and cardioprotective, properties. Its versatility extends to managing diabetes, reducing hyperlipidemia, mitigating nephrotoxicity, promoting hair growth, and protecting against obesity-related inflammation. These findings highlight OA’s potential for diverse clinical applications.

## 3. Combination with Other Natural Compounds or Conventional Drugs

The synergistic combination of natural compounds or drugs holds profound therapeutic potential, presenting a promising route for enhanced efficacy and unlocking the possibility of improved treatment outcomes and novel avenues for addressing complex medical conditions [77,78,79]. Numerous studies have investigated the synergistic potential of combining OA with various natural compounds or drugs.

OA, maslinic acid (MA), and their combination were studied to enhance the anticancer effect. Apoptosis increased in all treated groups. MA induced a 1.74-fold increase in autophagosomal LC3-II formation, while MA-OA induced a 3.25-fold increase. The combination treatment upregulated genes such as *GSK3B*, *PTEN*, *CDKN1B*, and *FOXO3*, and downregulated *IGF1*, *PRKCB*, and *AKT3* genes. The results indicated the highest synergistic effect at the lowest dose with MA-OA, inducing apoptosis in cancer cells. Combining drugs may alleviate cancer cell resistance to treatment [80].

In another research OA, UA, and triterpenoids used in treating diseases, including tuberculosis, were incorporated into a metered-dose inhaler (MDI) to enhance lung-specific drug delivery for advanced tuberculosis treatment. In Wistar rats, the combination therapy increased the biological half-lives of UA (9.23 ± 0.104 h) and OA (8.93 ± 0.166 h) with no adverse effects on body weight or vital organs in the in vivo toxicity study. The AUC of UA and OA was observed as 26.48 ± 1.29 and 428.25 ± 1.87 ng h/mL, respectively. Histopathology revealed no abnormalities, and mild alterations in biochemical and hematological parameters did not impact overall health [81].

A study assessed the impact of UA, OA, and the cytostatic drug camptothecin-11 (CPT-11) on colorectal cancer cells. Results showed reduced cancer cell viability and migration, downregulation of the urokinase plasminogen activator and its receptor (uPA/uPAR)-dependent matrix metalloproteinases (MMP) pathway, and increased E-cadherin levels. Combining UA and OA, especially with CPT-11, enhanced anti-tumorigenic effects without a severe impact on normal cells [82]. A different study explored the hepatoprotective effect of a UA and OA mixture against antituberculosis drug-induced damage. This UA/OA mixture, obtained from Rosmarinus officinalis methanolic extract, was administered intragastrically to male Balb/C mice at 10 and 20 mg/kg for 60 days. It showed positive impacts on body weight, reduced hepatic enzyme levels (AST, ALT, and ALP), and decreased steatosis, similar to the positive control, silymarin. The 10 mg/kg dose demonstrated significant hepatoprotective effects, suggesting its efficacy in countering antituberculosis drug-induced damage over 60 days [83]. Another research study probed a novel combination of OA and metformin for type 2 diabetes therapy. In a four-week treatment of male mice, the combination significantly lowered blood glucose and insulin levels, improved liver pathology, and enhanced glycogen-synthesis-related mRNA expression. The therapy also positively influenced signaling pathways, indicating synergistic effects in improving diabetes symptoms [84]. In the antibacterial study assessing the synergistic combination of OA and UA, the testing on four pathogens revealed synergistic effects when combined with β-lactam antibiotics (ampicillin and oxacillin) against Pseudomonas aeruginosa, Staphylococcus aureus, Staphylococcus epidermidis, and Listeria monocytogenes. This suggests that OA and UA could be useful in combination with β-lactam antibiotics to combat infections caused by certain Gram-positive pathogens, addressing antibiotic resistance [85]. In another investigation, a carrier-free nano-sensitizer (OC) was developed using self-assembly technology, combining the anticancer drug OA and the photosensitizer chlorin e6. OC exhibited enhanced accumulation in cancer cells and strong tumor penetration. It demonstrated significant synergistic inhibitory effects under light and ultrasound irradiation, causing high reactive oxygen species (ROS) generation, mitochondrial membrane potential loss, apoptosis, and cell cycle arrest. In vivo studies confirmed the synergistic therapeutic effects of OC in a mouse model. The self-assembled OC holds potential for synergistic chemo/sono-photodynamic therapy in cancer treatment [86].

## 4. Preclinical and Clinical Studies

OA has undergone comprehensive preclinical investigations, and although clinical studies are limited, emerging research suggests its potential therapeutic benefits in various applications. Pharmaceutical research interest sparked a focus on measuring OA in human serum. A study was conducted regarding bioavailability enhancement of OA, where 22 participants received a single 30 mg dose of OA in functional olive oil. Results revealed OA’s max serum concentration (500–600 ng mL^−1^) and AUC_0−∞_ (2862.50 ± 174.50 ng h mL^−1^). OA associates with serum albumin and triglyceride-rich lipoproteins (TRL), inducing conformational changes in albumin. TRL efficiently incorporates OA, reaching the maximum concentration (140 ng mL^−1^) after 2–4 h, suggesting potential as carriers for high bioavailability in target tissues [87]. Another report evaluated the clinical safety and effectiveness of NG440, a phytochemical-based anti-inflammatory formula combining rho-iso-alpha acids from hops, rosemary, and OA. The research demonstrated a significant 50% reduction in pain among osteoarthritis patients using NG440. Multicenter trial results confirmed reduced pain scores in individuals with joint discomfort. Ex vivo clinical studies suggested that NG440’s pain relief may result from decreased inflammatory cytokine production, including lower prostaglandin E2 formation. Animal toxicity data indicated NG440’s safety at dosages ≤ 250 mg kg^−1^ day^−1^ for 21 days, and human trial data showed no adverse effects on cardiovascular and gastrointestinal markers affected by COX-2 inhibitors. NG440 emerged as a safe and effective alternative in areas traditionally using specific COX-2 inhibitors [88].

## 5. Challenges in OA Delivery

The effective delivery of OA faces challenges related to its poor aqueous solubility, limited bioavailability, and the need for innovative drug delivery systems to enhance its therapeutic efficacy. “Poorly water-soluble” lacks an official definition in regulatory literature. The BCS categorizes drugs as highly or lowly soluble: “A drug substance is classified as highly soluble if the highest single therapeutic dose is completely soluble in 250 mL or less of aqueous media over the pH range of 1.2–6.8 at 37 ± 1 °C” [89]. According to this definition, if the drug does not exhibit high solubility, it falls into the category of low solubility. An alternative perspective for formulation scientists defines solubility as follows: soluble if >10 mg/mL, poorly soluble if <1 mg/mL, with an intermediate range (1 mg/mL < x < 10 mg/mL) indicating a gradual transition [90]. OA is categorized as a Class IV compound in the BCS system, characterized by both low water solubility, around 1 μg/mL, and limited permeability (Papp = 1.1–1.3 × 10^−6^ cm/s) [3,20]. OA’s low aqueous solubility and limited permeability are due to the presence of nonpolar functional groups in its structure. The molecule consists of a pentacyclic triterpenoid structure with multiple hydrophobic regions, such as bulky ring structures. These hydrophobic characteristics make OA poorly soluble in water [3,31]. The poor water solubility of a substance is linked to its restricted oral bioavailability [91], as indicated by a study conducted by Jeong, which reported an absolute oral bioavailability of 0.7% with oral doses of 25 and 50 mg/kg of OA in rats. The extremely low oral bioavailability of OA may be attributed to inadequate absorption and substantial metabolic clearance [20]. Pharmaceutical compounds characterized by low solubility present a great risk of challenges during drug innovation and development. Solubility significantly impacts key parameters, such as pharmacokinetics, pharmacodynamics, drug distribution, protein binding, and absorption [92]. In the realm of pharmaceutical research, exploring effective drug delivery systems is crucial for optimizing the therapeutic potential of bioactive compounds. Addressing these challenges in OA administration requires innovative formulation strategies aimed at improving their solubility, bioavailability, and overall therapeutic efficacy to evaluate the full therapeutic potential of these molecules in different clinical applications.

In the contemporary era of technological advancement, novel DDS have emerged as a gateway to enhancing the bioavailability of herbal drugs [93]. These innovative formulations offer significant advantages over traditional plant active formulations, such as increased solubility, enhanced bioavailability, mitigation of toxicity risks, increased pharmacological activity and intracellular uptake, enhancement of pharmacokinetics and biodistribution, improved distribution to tissue macrophages, sustained release, and protection against physical and chemical degradation [94].

**Figure 3:**
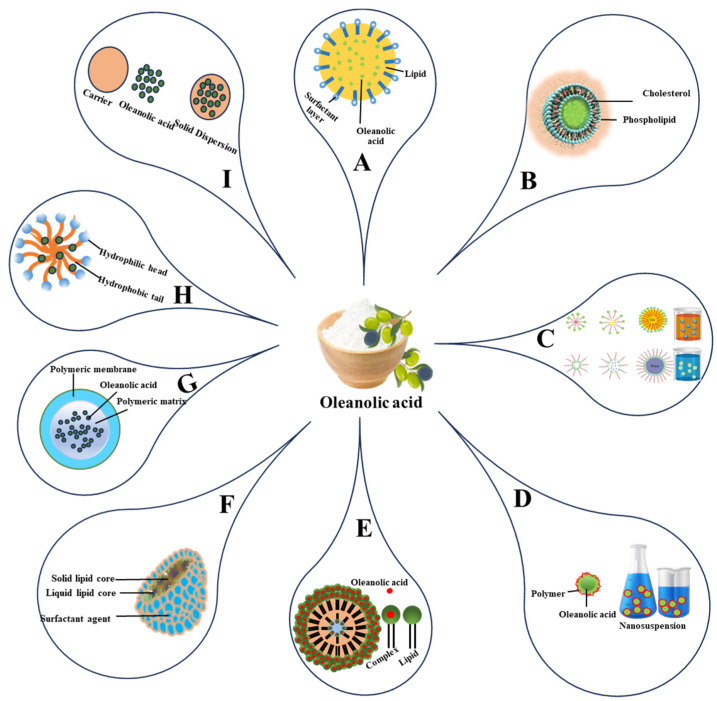
Illustration of oleanolic acid delivery using various lipid-based systems and nanocarriers: (**A**) solid lipid nanoparticle, (**B**) liposomes, (**C**) microemulsions, (**D**) nanosuspension, (**E**) phospholipid complexes, (**F**) nanostructured lipid carriers, (**G**) nanoparticles, (**H**) micelles, and (**I**) solid dispersion.

The following discussion explores various approaches and advances in OA delivery systems in different therapeutic contexts. Figure 3 provides a visual overview of OA delivery systems applied to OA formulation.

### 5.1. Lipid-Based Delivery Systems

In recent years, much attention has been devoted to using lipid-based drug delivery systems (LBDDS) to improve the oral bioavailability of poorly water-soluble pharmaceuticals, and to achieve precise and time-regulated drug release [95,96]. LBDDS effectively handle drugs with low water solubility, offering size-dependent properties and prominent advantages, such as high biocompatibility and versatility. Commercially viable for various delivery routes, these systems can be tailored to meet specific product requirements, making them attractive for pharmaceuticals, vaccines, diagnostics, and nutraceuticals [97]. LBDDs have gained importance for enhancing the solubility and bioavailability of poorly water-soluble drugs [98]. In particular, oral absorption of lipophilic compounds can be significantly improved using these systems. LBDDs, such as micro- and nano-emulsions, solid lipid nanoparticles, and nanostructured lipid carriers, represent a successful example of nanoformulations applied to overcome the limitations of natural compounds [99,100,101,102,103,104,105]. Several research activities have also been performed to improve the solubility and dissolution profile of OA (Table 1 and Table 2).

#### 5.1.1. Solid Lipid Nanoparticles and Nanostructured Lipid Carriers

Solid lipid nanoparticles (SLN; Table 1) provide controlled drug release, targeted delivery with low toxicity, increased stability, and high drug loading. SLN are made of lipids, solid at both body and room temperatures. These lipids are dispersed in an aqueous medium and usually stabilized by a surfactant that covers the solid core [106,107].

A study was conducted to produce OA-SLN employing the film-ultrasound dispersion technique [108]. Another study aimed to produce OA-SLN using the film-ultrasonic wave dispersion technique. Optimal conditions were determined through an orthogonal test based on encapsulation efficiency. Results showed that a 40 min ultrasonic wave duration, a 1:8 ratio of OA to phospholipids, and 15 mL of 60 g/L mannitol yielded nanoparticles with a diameter distribution of 75 ± 20.3 nm and encapsulation efficiency exceeding 97.81% [109]. Similarly, OA-SLN were prepared using an improved emulsion-solvent evaporation method. In vitro release testing revealed a slow-release rate of OA at 4.88% per hour, consistent with the zero-order release model. Additionally, OA-SLN demonstrated stability in artificial gastric and intestinal juices [110].

The evolution from SLN to nanostructured lipid carriers (NLC; Table 1) is a significant advancement in DDS, with both platforms demonstrating remarkable versatility and potential in pharmaceutical applications. The NLC are advanced drug delivery systems with a liquid and solid matrix at room temperature, composed of biocompatible lipids and surfactants. They have gained regulatory approval and witnessed rapid market growth, with around 30 commercial preparations available. NLC offer superior advantages over other colloidal carriers, including an enhanced drug loading capacity and modulation of drug release [111]. A study was conducted to develop NLC loaded with OA and gentiopicrin, using glycerin monostearate, oleic acid, and Poloxamer 188 to produce optimized NLC. Notably, a sustained release profile was observed. Co-loaded NLC extended drug concentrations in plasma, offering protective effects against hepatic injury compared to individual loading. The study confirmed the successful simultaneous loading of OA and gentiopicrin into NLC for enhanced therapeutic benefits [112]. In a similar study, OA-NLC, initially composed of soy lecithin (LC), tristearin (TS), and palmitic acid (PA) at a molar ratio of 2:2:1, was improved by partially substituting LC with an ion-pair amphiphile (NLCIPA). The optimal LC/IPA ratios for NLCIPA formulations were determined to be 2:3, 3:7, and 1:4. NLCIPA showed higher stability than conventional NLC, with 30 mol% LC considered optimum. OA-loaded NLCIPA exhibited enhanced drug accumulation and sustained release compared to NLC. In cytotoxicity studies on cancer cell lines, OA-loaded NLCIPA demonstrated superior efficacy over NLC, making NLCIPA a promising alternative for enhancing the therapeutic efficacy of OA as an anticancer drug [113].

#### 5.1.2. Micro- and Nano-Emulsions

**Figure 4:**
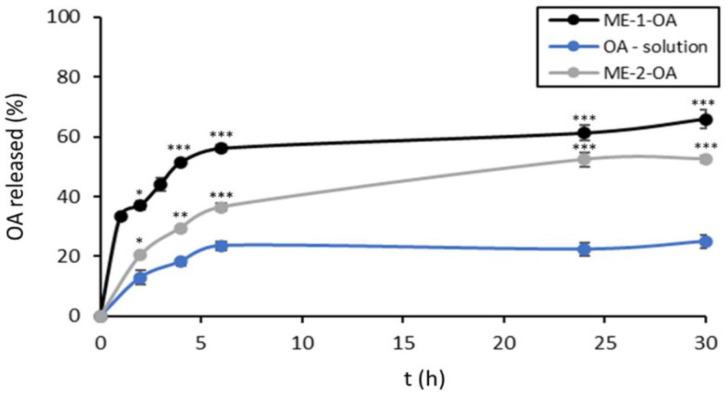
In vitro release profiles of OA from solution, ME-1 and ME-2, in EtOH:PBS (30:70). Values are reported as mean ± SD of three independent experiments. Tukey’s test (*n* = 3): * *p* < 0.05, ** *p* < 0.01, and *** *p* < 0.001 vs. OA solution at the corresponding times. Reproduced with permission from [44], MDPI, 2022.

Microemulsions (ME) are transparent, thermodynamically homogeneous dispersions of two immiscible liquids stabilized by an interfacial film of surfactants. They require very low energy to be formulated, as they form spontaneously when aqueous, oily, and amphiphilic components are brought into contact, as well as having a lower production cost than nano-emulsions. They offer advantages for drug delivery, including easy formation, stability, and improved solubilization of drugs [114,115,116]. Some applications are summarized in Table 1. Building on the advantages of ME as an optimal choice for orally delivering olive extract [117], a recent study focused on pentacyclic-triterpenes-enriched extract (EXT), with the main component being OA. The study developed a ME loaded with EXT for oral administration. The ME-EXT showed increased EXT solubility, stability over two months, prolonged release of triterpenes, improved permeability, and enhanced inhibition of intracellular lipid accumulation in human hepatocarcinoma cells (HepG2) [118]. In a subsequent study, two microemulsions (ME-1 and ME-2) were formulated to enhance OA’s solubility and intestinal permeability. Both MEs increased OA solubility significantly. An in vitro intestinal permeability assay demonstrated an enhanced passive permeability for both ME formulations compared to free OA. Moreover, the release of OA was gradual and sustained, with the percentage reaching 60% for ME-1 and 51% for ME-2 after 8 h, as shown in Figure 4. The study also suggested that both MEs enhanced OA’s protective action against LPS-induced oxidative stress in murine macrophages, indicating potential clinical applications for improving OA oral bioavailability [44]. A self-micro-emulsifying drug delivery system (SMEDDS) was developed to enhance the solubility and oral bioavailability of OA. Compared to conventional tablets, SMEDDS showed a 5.07-fold increase in oral bioavailability in rats [119].

Nano-emulsions (NE) are composed of extremely small droplets, usually oil dispersed in water, with sizes below 300 nm. They can be prepared using lower concentrations of surfactants with respect to ME. They are fine dispersion systems not in equilibrium, with a spontaneous tendency to separate into constituent phases. They can have a relatively high kinetic stability due to their very small size, essentially as a result of considerable steric stabilization between the drops. Despite being in a non-equilibrium state, they exhibit slow destabilization kinetics, making them kinetically stable due to their tiny size, which prevents droplet flocculation and coalescence, primarily influenced by Ostwald ripening [120,121,122,123,124]. NE protects the active ingredient from degradation and extends its bioavailability [124]. The applications of NE to OA delivery are reported in Table 1. In a study, a self-nano-emulsified drug delivery system (SNEDDS) was developed for OA oral delivery. Optimized formulations demonstrated significantly improved dissolution and oral bioavailability compared to a commercial tablet [125]. Similarly, another study designed and optimized a NE for dermal delivery of OA and UA, natural or synthetic, using castor oil, labrasol, transcutol-P, and propylene glycol. The selected Smix ratio (4:1) produced two NEs with mean droplet sizes below 600 nm. Both formulations, exhibiting similar permeation parameters for local action, proved non-toxic and non-irritating in vivo. Additional parameters assessed included the mean dissolution time (MDT) and dissolution efficiency (DE). MDT values were 32.71 ± 0.88 h and 32.41 ± 1.86 h for NM-OA/UA NE and SM-OA/UA NE, respectively. Similarly, DE values were 56.71 ± 1.17% and 56.78 ± 2.48%, respectively. The NE loaded with OA and UA demonstrated superior anti-inflammatory efficacy compared to the synthetic counterpart, accentuating the potential of o/w NE for effective dermal delivery of OA and UA [24].

#### 5.1.3. Liposomes

**Figure 5:**
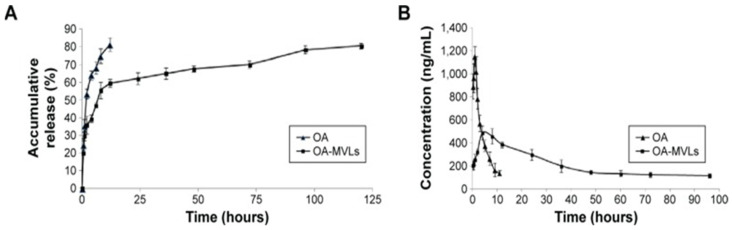
Drug release characterization both in vitro (**A**) and in vivo (**B**) from the formulation of OA-MVLs. Reproduced with permission from [132], Dovepress, 2016.

Liposomes are sphere-shaped vesicles comprising one or more layers of phospholipids, significantly impacting DDS. They have become indispensable tools in various scientific fields and represent advanced drug delivery systems, with several formulations in clinical use. These lipid vesicles have enhanced the formulation of potent drugs, aiming to minimize toxicity and improve accumulation at the intended target site [126,127]. Different studies were conducted for the development of OA liposomes (Table 2). Galactosylated chitosan-modified liposomes (GC@Lipo) were developed to address the challenge of non-specific drug distribution in hepatocellular carcinoma (HCC) treatment. These liposomes selectively bind to the asialoglycoprotein receptor (ASGPR) on HCC cell surfaces. The study showed that GC@Lipo significantly improved the antitumor efficacy of OA by enabling targeted drug delivery to hepatocytes. In mouse Hepa1-6 cells, OA-loaded GC@Lipo inhibited migration and proliferation, upregulating E-cadherin and downregulating N-cadherin, vimentin, and Anexelekto compared to free OA solution and OA-loaded liposomes. In an axillary tumor xenograft mouse model, OA-loaded GC@Lipo reduced tumor progression, concentrating in hepatocytes [128]. An investigation was carried out to address the health risks posed by airborne fine particulate matter (PM2.5), particularly in manual laborers who are exposed to both PM2.5 and alcohol. The combination of PM2.5 exposure and alcohol consumption can exacerbate liver damage and lead to conditions such as liver fibrosis. To mitigate these risks, OA-liposomal nanovesicles (OA-Lipo) were developed, exhibiting reduced cytotoxicity and anti-inflammatory effects. In a mouse model, OA-Lipo effectively alleviated PM2.5 and alcohol-induced liver fibrosis, suggesting their potential for treating environmental and alcohol-related liver injuries [129]. Likewise, a study was intended to improve the solubility and oral bioavailability of OA using polyvinylpyrrolidone (PVP)-modified liposomes. These liposomes, composed of soybean lecithin, cholesterol, PVP-K30 coating, and sodium deoxycholate, were prepared to enhance drug solubility. Characterization showed spherical particles with a high encapsulation efficiency (>90%). Compared to commercial tablets, the liposomal formulation increased OA’s maximum plasma concentration by 6.90-fold in rats, indicating a relative bioavailability of 607.9% [130]. Similarly, nanoliposomes of OA were prepared using the modified ethanol injection method. These liposomes featured a hydrophobic OA core, an amphiphilic soybean lecithin monolayer, and a protective hydrophilic polyethylene glycol (PEG) coating. Results demonstrated slower OA release from PEGylated liposomes, potentially reducing drug toxicity. Surface-modified liposomes exhibited enhanced inhibition of HeLa cells, and the liposomal formulations displayed improved in vitro stability compared to native OA. A suggested mass ratio of OA to PEG is 1:1, proposing these liposomes as an effective antitumor drug delivery system, particularly beneficial in cancer therapy [131]. In a similar manner, a study aimed to develop multivesicular liposomes (MVLs) encapsulating OA to address its poor solubility and enhance its antitumor efficacy against hepatocellular carcinoma. Using a double-emulsion method, optimized OA-MVLs were synthesized and characterized. In vitro studies demonstrated sustained drug release, as shown in Figure 5A, and effective inhibition of HepG2 cell growth. In vivo, OA-MVLs exhibited prolonged circulation compared to the OA solution (Figure 5B), no observed toxicity at medium doses, and suppressed murine H22 hepatoma growth, extending survival in tumor-bearing mice [132].

**Table 1:** OA-loading SLN, NLC, ME, and NE: characteristics and performance evaluation. ↑: increase; ↓: decrease.

DDS	Components	Chemical and Physical Parameters					In Vitro Release	Solubility (mg/mL)	Permeability (Pe)	Activity and/or Bioavailability	References
		EE	DL	Size (nm)	Z-Potential (mV)	PDI					
SLN and NLC	OA and lipid	94.2 ± 3.9%	4.7 ± 0.2%	104.5 ± 11.7	−25.5 ± 1.8	-	4.88% per hour	-	-	-	[110]
	OA and gentiopicrin, glycerin monostearate, oleic acid, Poloxamer 188	48.3 ± 2.7%	8.1 ± 0.4%	111.0 ± 1.6	−23.8 ± 0.4	0.287 ± 0.01	70% after 10 h	-	-	1520.7 ± 25.3 (μg min/mL) i/v route ↓ ALT ↓ AST	[112]
	NLC: Soy lecithin, tristearin, palmitic acid (2:2:1) IPA: sodium dodecyl sulphate, hexadecyltrimethylammonium bromide (1:1) NLC_IPA_ = Lecithin + IPA (2:3, 3:7, 1:4) OA NLC_IPA_ = OA-NLC_IPA_	NLC: 75% NLC_IPA_ (2:3): 85% NLC_IPA_ (3:7): 90% NLC_IPA_ (1:4): 90%	NLC:8% NLC_IPA_ (2:3): 8.7% NLC_IPA_ (3:7): 9.2% NLC_IPA_ (1:4): 9.2%	NLC (250–258) NLC_IPA_ (139–150) OA-NLC_IPA_ (140–160) OA-NLC (260–265)	NLC (−10 to −12) NLC_IPA_ (−5 to −9) OA-NLC_IPA_ (−4 to −8) OA-NLC (−9 to −9.5)	0.3–0.5	NLC = 80% NLC_IPA_ (2:3): 62% NLC_IPA_ (3:7): 42% NLC_IPA_ (1:4): 60% (after 80 h)	-	-	Anticancer activity in HepG2, Huh-7, HCT-116, OA-NLC_IPA_ ˃˃˃OA-NLC	[113]
ME	Smix: Transcutol HP, TPGS (9:1) Oil = Capryol 90 Smix/Oil: (9:1)	-	2 mg/ mL	107.8 ± 1.5	−20.5 ± 0.4	0.24 ± 0.02	73.2 ± 1.7% after 24 h	2	1.02 × 10^−6^ ± 1.48 × 10^−7^ cm/s after 6 h	↓ HepG2-induced intracellular lipid level	[118]
	ME-1-OA (Capmul PG-8/NF Transcutol HP, Tween 20, water, 6:17:37:40) ME-2-OA (Nigella oil/ isopropyl myristate (1:1), Transcutol HP, Cremophor EL, water, 4:30:16:50)	-	ME-1-OA (1 mg/mL) ME-2-OA (3 mg/mL)	ME-1-OA (93.0 ± 3.4) ME-2-OA (17.62 ± 0.23)	ME-1-OA (−3.32 ± 0.02) ME-2-OA (−11.63 ± 0.01)	ME-1-OA (0.20 ± 0.04) ME-2-OA (0.20 ± 0.07)	-	ME-1-OA (1000-fold) ME-2-OA (3000-fold)	ME-1-OA 5.70 ± 0.01 × 10^−6^ cm/s ME-2-OA 4.74 ± 0.04 × 10^−5^ cm/s	↑ bioactivity of OA against LPS-induced oxidative stress in RAW 264.7 murine	[44]
	Ethyl oleate, Cremophor EL, Ethanol (50:35:15)	-	13.19 ± 0.33	49.7	-	-	˃85% in 60 h	13.19 ± 0.33 mg/g	-	↑ 1740.1 (ng h/mL) 5.07-fold	[119]
NE	Formulation-I Sefsol 218/Cremophor EL/Labrasol (50:25:25), Formulation-II Sefsol 218/Cremophor EL/Labrasol/Transcutol P (50:22.5:22.5:5), Formulation-III Sefsol 218/Cremophor EL/Labrasol/Transcutol P (50:20:20:10), Formulation-IV Sefsol 218/Cremophor EL/Labrasol/Transcutol P (50:17.5:17.5:15)	-	-	Formulation-I 38.4 ± 0.2 Formulation-II 46.4 ± 0.5 Formulation-III 75.3 ± 0.3 Formulation-IV 110.4 ± 0.6	-	Formulation-I 0.055 Formulation-II 0.120 Formulation-III 0.238 Formulation-IV 0.258	˃75%	-	-	-	[125]
	Natural mixture (NM-OA/UA) and synthetic mixture (SM-OA/UA) castor oil (20%), and propylene glycol (20%) Smix (59.80%): (labrasol, transcutol-P) (4:1)	-	0.2% (*w*/*w*)	NM-OA/UA (200.9–590.5) SM-OA/UA (139.7–450.9)	-	NM-OA/UA (0.25 to 0.73); SM-OA/UA (0.18 to 0.66)	NM-OA/UA DS (56.7 ± 1.2%) SM-OA/UA DS (56.8 ± 2.5%)	-	NM-OA/UA %A_48_, (6.41–7.39), SM-OA/UA %A_48_, (4.96–6.24)	↑ In vivo anti-inflammatory activity	[133]

Abbreviations: NE: nano-emulsion, ME: microemulsion, SLN: solid lipid nanoparticles, NLC: nanostructured lipid carriers, IPA: ion-pair amphiphile; %A_48_: amount of OA/UA permeated at 48 h, OA: oleanolic acid, UA: ursolic acid, ALT: alanine aminotransferase, AST: aspartate aminotransferase.

**Table 2:** OA-loading liposomes, micelles, and nanosuspensions: characteristics and performance evaluation. ↑: increase; ↓: decrease.

DDS	Components	Parameters of Formulations					In Vitro Release	Solubility (mg/mL)	Permeability (Pe)	Activity and/or Bioavailability	References
		EE	DL	Size (nm)	Z-Potential (mV)	PdI					
Liposomes	DSPC: 8.7 mg, cholesterol: 2.9 mg OA: 6.85 mg	79%	-	353 ± 140	-	-	54% release in 24 h	-	-	↓ OA cytotoxicity ↑ hepatoprotective effect	[129]
	Soybean phospholipid: 10 mg OA: 1 mg, cholesterol: 1 mg, sodium deoxycholate: 1 mg	93.9%		179.4 ± 3.15	−28.8 ± 1.72	0.159 ± 0.061	-	-	-	Increased bioavailability of 607.9%	[130]
	soybean phosphatidylcholine, cholesterol, PEG-2000	63–98%	-	<200	-	-	slower release	-	-	reduce drug toxicity	[131]
	soybean lecithin 80 mg, cholesterol 45.6 mg, triolein 20 mg, stearic acid 1 mg, OA 16 mg, aqueous solution Tween-80, polyvinyl alcohol	82.3 ± 0.61%	-	Average: 11.57 μm	−13.35 mV	0.21	80% release in 120 h	-	-	suppressed the growth of murine H22 hepatoma AUC_0–∞_ (h ng mL^−1^) 26,131.37	[132]
Micelles	OA: 50 mg Polygalacturonic acid: 50 mg	76.59 ± 4%	53.94 ± 3%	200.0	−44.7	-	pH2: 7.5% pH6.8: 13% pH7.4: 18.5% SGF: no release SIF: approx. 12%	-	-	↑ Increased insulin sensitivity ↓ the blood glucose C_max_: 12.52-fold increased at 6 h	[134]
	TPGS 120: mg Pluronic P105: 80 mg OA: 7 mg	93.6 ± 0.05%	3.5%	95.7 ± 3.6	−8.6 ± 0.4	0.13 ± 0.03	~80% free OA, ~40% OA- micelles at 24 h	-	-	↑ antitumor effect in A549 and PC-9 cells.	[135]
	Polymeric micelles-G *w*/*w*% (OA: 0.05; Capryol 90: 2; Poloxamer 407: 6) Polymeric micelles-H *w*/*w* (OA: 0.05; Capryol 90: 2; Poloxamer 407: 7)	Polymeric micelles-G: 98.26 ± 0.17% Polymeric micelles-H: 99.18 ± 1.06%	-	Polymeric micelles-G: 80.4 Polymeric micelles-H: 57.0	-	-	-	-	Polymeric micelles-H: 29.49 ± 4.00% Polymeric micelles-G: 21.39 ± 5.91% at 24 h	alleviated skin wrinkles	[136]
Nanosuspensions	OA and Tween 80	-	-	284.9	−27.6 ± 7.2	0.22	95% 2 h	0.026	-	↑ hepatoprotective effect	[137]
	SEL:SEP at 4:1 *w*/*w* SE:OA at 2:1 *w*/*w*	45.38 ± 1.81	-	96.60 ± 2.30	-	0.41 ± 0.03	100% 2 h	1.89 ± 0.08	-	↓ proliferation rate of A549 cell lines Bioavailability: 7 times increased	[138]
	lyophilized whole whey, lactose monohydrate, OA	-	-	60.3 ± 7.4	−14.66	0.21	97% 2 h	-	-	Improved antidiarrheal effect	[139]

Abbreviations: OA: oleanolic acid, DSPC: 1,2-distearoyl-sn-glycero-3-phosphocholine, TPGS: vitamin E-D-α-tocopheryl polyethylene glycol succinate, SEL: sucrose monolaurate, SEP: sucrose monopalmitate.

### 5.2. Micelles

**Figure 6:**
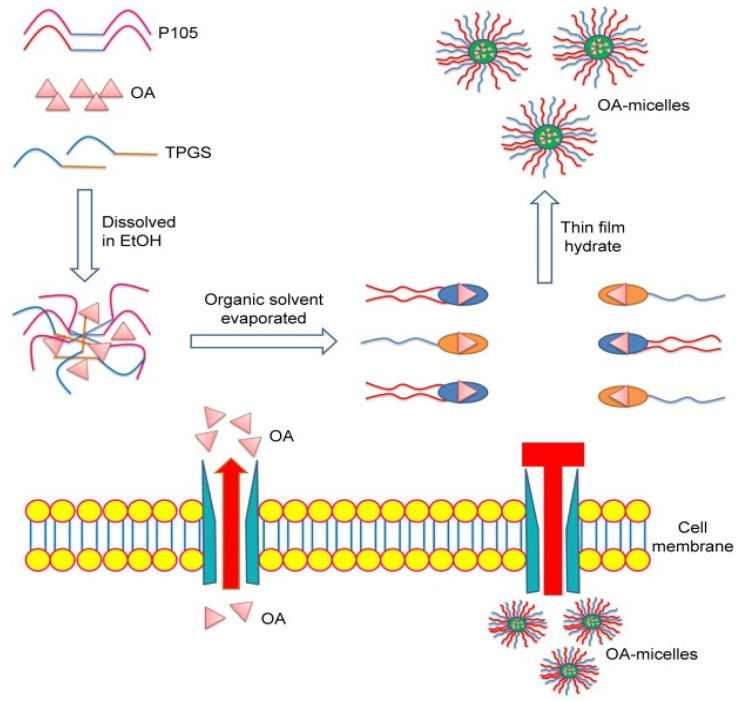
Preparation scheme of OA-micelles via the self-assembly method and different phenomena between OA-micelles and free OA in the cell. EtOH, ethyl alcohol; OA, oleanolic acid; P105, Pluronic P105; TPGS, d-α-tocopheryl polyethylene glycol succinate. Reproduced with permission from [135], Dovepress, 2016.

Micelles, formed by the noncovalent aggregation of surfactant monomers, exhibit diverse shapes, such as spheres, cylinders, or planar structures. The ability of micelles to adopt various shapes based on surfactant chemistry and solution conditions influences the design and structure of micelle-based delivery systems [140]. Micelle-based delivery systems offer remarkable versatility in transporting a range of payloads, such as drugs, proteins, peptides, DNA, SiRNA, and more. These nanocarriers predominantly exhibit a spherical structure, which provides exceptional physical stability attributed to their minimal surface energy [141]. The micelles typically exhibit sizes ranging from 10 to 100 nm [142]. There has been significant focus on the potential applications of micelles within three primary domains of drug delivery: enhancing drug solubility, regulating drug release, and targeting specific sites for drug delivery (Table 2) [143]. Researchers have developed a natural plant-derived polymeric micelle system for oral insulin resistance treatment in type II diabetes (T2D). They have utilized plant-derived OA and poly-galacturonic acid (PGA) sourced from citrus pectin. These blank micelles were then loaded with OA (PGA-OA), serving as both a component of the micelles and as the drug. The formulation showed improved insulin sensitivity without side effects in HepG2 cells in vitro and in a T2D rat model in vivo. The micelles exhibited enhanced absorption, crossed gastrointestinal barriers, and maintained a prolonged plasma drug concentration for up to 24 h. In a T2D rat model, the formulation effectively reversed insulin resistance, providing sustained glucose control even after drug withdrawal. The C_max_ increased by 12.52-fold at 6 h by micelles. They also enhanced insulin sensitivity and significantly decreased blood glucose levels. The molecular mechanism involves promoting the IRS-1/PI3K/Akt signaling pathway and inhibiting PTP1B enzyme activity [134]. Similarly, vitamin E-D-α-tocopheryl polyethylene glycol succinate (TPGS) and Pluronic P105 enhanced the solubility of OA, forming mixed micelles. These micelles showed slower OA release, increased uptake by lung cancer cells, and effective accumulation in tumors in vivo. OA-micelle-treated mice exhibited smaller tumors and higher pro-apoptotic protein expression, demonstrating superior efficacy compared to free OA. In vitro studies confirmed lower inhibitory concentrations, higher apoptotic rates, and suppressed migration and invasion in OA-micelle-treated cells. To supplement these findings, Figure 6 illustrates the preparation scheme of OA-micelles via self-assembly and the differences between OA-micelles and free OA in cellular environments [135]. OA, traditionally a secondary ingredient in cosmetics, was studied as a primary active compound for reducing wrinkles. Polymeric micelles were prepared using Capryol 90 and Poloxamer 407, resulting in sizes below 100 nm. The inner core of micelles encapsulated nearly 100% of the OA. The formulation was applied around the eyes of 23 female subjects for 8 weeks, and the micelles demonstrated higher skin permeation and stability. Results showed significant improvement in skin parameters and visual evaluation scores without irritation [136].

### 5.3. Nanosuspensions

Nanosuspension (NS), with its submicron particle size and distinct physicochemical properties, has the potential ability to tackle many formulation and drug delivery issues typically associated with poorly water- and lipid-soluble drugs [144]. While there are various alternative technologies available, nanosuspensions are particularly well-suited for drugs exhibiting high crystal energy, resulting in their insolubility in both lipid and aqueous vehicles [145]. The applications of NS technology to OA delivery are shown in Table 2. A study employed the nanoprecipitation method to prepare OA NS, resulting in a six-fold increase in saturation solubility at 25 °C (25.72 µg/mL vs. 4.37 µg/mL). In vitro drug release experiments demonstrated that lyophilized NS exhibited a faster dissolution rate compared to coarse drug powder, with a nearly 95% release of OA within 2 h [137]. Similarly, a study was conducted to develop sucrose ester (SE)-stabilized OA-NS for enhanced delivery. SEOA NS were prepared via O/W emulsion and organic solvent evaporation methods. In particular, SEOA 4121 NS (SEL (sucrose monolaurate):SEP (sucrose monopalmitate) 4:1 *w*/*w*; SE: OA at 2:1 *w*/*w*) produced the greatest increase in saturation solubility (1.89 mg/mL vs. 3.43 μg/mL), dissolution rate, cytotoxicity, and bioavailability. Preliminary studies indicated that cellular uptake of SEOA NS by A549 cells was temperature-, concentration-, and time-dependent [138]. In the same way, a study was designed to develop a lyophilized OA-NS using whole whey. In vivo tests in rats showed that OA-NS had better dissolution rates than free OA, possibly due to a shift from crystalline to amorphous form. The whole-whey-stabilized OA-NS offers a novel approach for improving dissolution rates and long-term stability, potentially benefiting other poorly water-soluble drugs [139].

### 5.4. Nanoparticles

**Figure 7:**
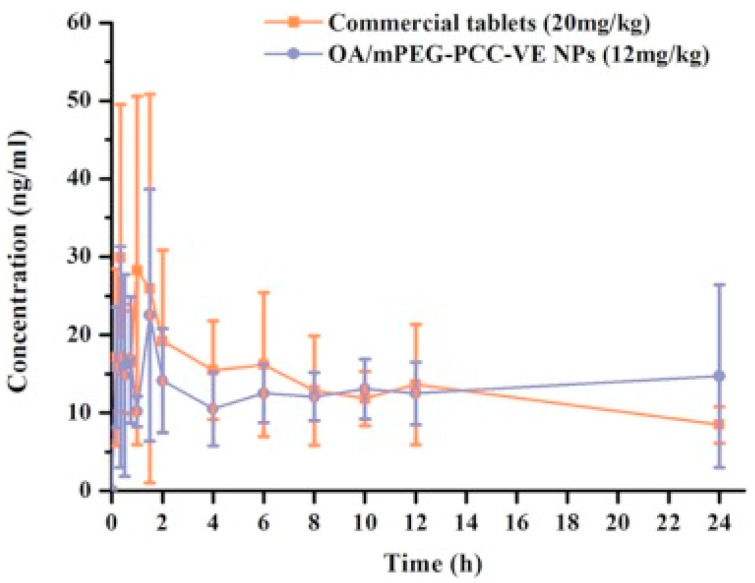
Mean plasma concentration–time curves of OA in rats after oral administration of commercial tablets and OA/mPEG-PCC-VE NPs (data are mean ± SD, *n* = 5). Reproduced with permission from [153], Elsevier, 2017.

Nanoparticles (NP), with their small size and unique properties, show great promise as drug carriers, enabling precise targeting and enhanced effectiveness in biomedical applications [146]. In the literature, different examples confirmed their effectiveness for OA delivery (Table 3). A study employed amphiphilic carboxylated cellulose-graft-Poly(L-lactide) (CC-g-PLLA) copolymers, particularly PLLA with a degree of substitution of 2.03, self-assembled into nanoparticles for efficient OA delivery. These nanoparticles, with a small size (196.82 ± 9.14 nm) and high drug loading efficiency (24.76 ± 0.58%), effectively solubilized OA (16.9 mg/mL) and demonstrated prolonged drug release (120 h). In vitro and in vivo studies confirmed their maintained cytotoxicity to cancer cells (4T1 and MCF-7) and high antitumor efficiency, establishing the potential of these amphiphilic CC-g-PLLA copolymer nanoparticles as a good drug delivery system [147]. Another study was conducted to synthesize cetuximab (CTX)-functionalized albumin nanoparticles (ALB-NP) containing OA for targeted therapy of epidermal growth factor receptor (EGFR) in lung cancer. CTX is a monoclonal antibody specifically targeting the EGFR, and albumin is used as a carrier. Molecular docking determined the suitable nanocarrier, and various physicochemical parameters were analyzed. CTX-conjugated ALB-NP exhibited higher cellular uptake and significantly lower IC50 values in A549 cells compared to non-targeted ALB-NP. The nanoparticles induced apoptosis, blocked the cell cycle, and demonstrated biocompatibility in hemocompatibility, histopathology, and lung safety studies. In vivo imaging confirmed targeted delivery to lung cancer, suggesting CTX-OA-ALB-NP as potential carriers of OA for effective and targeted lung carcinoma therapy [148]. A research study investigated the use of polysaccharides from *Actium lappa* polysaccharide (ALP) as natural materials to synthesize nanoparticles for delivering poorly water-soluble OA and UA. The encapsulation process resulted in a more amorphous state with enhanced anti-inflammatory activity in a zebrafish model, suggesting that ALP-NP serve as natural carriers for hydrophobic bioactive molecules [149]. Similarly, OA-NP were prepared using polylactic co-glycolic acid (PLGA) and TPGS. The NP fabricated through the emulsion solvent evaporation method exhibited successful cellular uptake. In vitro studies demonstrated sustained drug release, with the highest loading at 11.08 ± 0.35% and 85.66 ± 0.56% release over 30 days. The nanoformulation effectively suppressed the amastigote burden in the spleen of BALB/c mice by 98.82 ± 1.92% in vivo, making it a promising carrier for OA against Leishmania donovani, exceeding the efficacy of pure OA for visceral leishmaniasis treatment [150]. In a study, the researchers developed an OA-loaded lactoferrin nano-delivery system, enhancing in vitro dissolution and oral absorption. The NP exhibited a significant increase in in vivo bioavailability compared to free drug administration in rats. The lactoferrin nano-delivery system proves to be a promising strategy for improving the oral absorption and bioavailability of poorly soluble drugs [151]. Similarly, in another study, OA was encapsulated into NP and liposomes. Optimized NP formulations resulted in a diameter of 150 nm. The liposomes and pro-liposome consisted in the hydrophobic core of OA, surrounded by a soybean lecithin monolayer and a hydrophilic PEG coating, with diameters ranging from 110 to 140 nm. The NP by precipitation and liposome methods achieved high encapsulation efficiencies of 86.7% and 92.6%, respectively. Both nanoparticle types exhibited increased stability at 4 °C across various conditions [152]. Zhang et al. developed a novel vitamin E-modified aliphatic polycarbonate-based polymeric nanoparticle (mPEG-PCC-VE NP) delivery system to improve oral absorption of OA. The OA-NP, with a uniform size of around 170 nm and high drug loading (8.9%), demonstrated enhanced in vitro dissolution in an alkaline medium. In rat studies, these NP significantly improved permeability throughout the intestinal tract, particularly in the duodenum and colon. In vivo pharmacokinetics showed a 1.5-fold higher bioavailability for OA/mPEG-PCC-VE NP compared to commercial OA tablets (Figure 7). This emphasizes the potential of mPEG-PCC-VE NP for effective oral OA delivery [153].

**Table 3:** OA-loading nanoparticles: characteristics and performance evaluation. ↑: increase.

DDS	Components	Parameters of Formulations					In Vitro Release	Permeability (Pe)	Activity and/or Bioavailability	References
		EE	DL	Size (nm)	Z-Potential (mV)	PDI				
NP	carboxylated cellulose-graft-Poly(L-lactide) (CC-g-PLLA) copolymers	-	24.76 ± 0.58	196.82 ± 9.14	−0.23 ± 0.12	-	90.5% at pH 6, 80.0% at pH 7.2, over 120 h	-	high efficiency against 4T1 cells and MCF-7	[147]
	ALB CTX	OA-ALB-NPs: 82.2 ± 3.9% CTX-OA-ALB-NPs: 78.08 ± 2.3%	-	OA-ALB-NPs: 171 ± 4.8 CTX-OA-ALB-NPs: 180 ± 3.7	OA-ALB-NPs: −30.5 ± 2.8 CTX-OA-ALB-NPs: −33.3 ± 3.4	OA-ALB-NPs 0.14 ± 0.07 CTX-OA-ALB-NPs 0.26 ± 0.03	OA-ALB-NPs pH 5.5: 95% OA-ALB-NPs pH 7.4: 75% CTX-OA-ALB-NPs pH 5.5: 85% CTX-OA-ALB-NPs pH 7.4: 70%	-	↑ A549 cells’ susceptibility AUC total (ng.h/mL) OA 31,195.8 ± 1127.2 OA-ALB-NPs 68,557.01 ± 1424.36 CTX-OA-ALB-NPs 73,592.7 ± 1148.57	[148]
	ALPs: 50 mg UA: 1 mg OA: 1 mg	48.98 ± 3.77%	0.96 ± 0.07	199.1	–7.15	0.35–0.40	-	-	enhanced anti-inflammatory effect	[149]
	PLGA, TPGS F1 = 1:5 Drug: polymer F2 = 1:10 Drug: polymer	F1: 66.47 ± 2.12 F2: 40.92 ± 2.37	F1: 11.08 ± 0.35 F2: 3.72 ± 0.22	F1 = 377.9 ± 5.7 F2 = 233.9 ± 2.4	F1: −15.6 ± 1.4 F2: −10.7 ± 1.7	F1: 0.235 F2: 0.415	F1: 85.66 ± 0.56% F2: 70.34 ± 1.15% 30 days	-	Enhanced potency against visceral leishmaniasis	[150]
	OA: Lactoferrin 1:6	92.59 ± 3.24%	12.44 ± 2.5	202.2 ± 8.3	+27.1 ± 0.32	0.15 ± 0.03	97.31 ± 2.04% in 20 min	-	↑ 340.59% AUC_last_ (ng h/mL) 126.53 ± 22.00	[151]
	soybean lecithin, PEG NP-A precipitation method, NP-B: liposomes	NP-A: 86.7% NP-B: 92.6%	-	NP-A: 150 NP-B: 110–140	-	-	-	-	-	[152]
	Vitamin E-modified aliphatic polycarbonate (mPEG-PCC-VE)	71%	8.9	165.06 ± 1.08	-	0.13 ± 0.06	OA/mPEG-PCC-VE NPs 78% in 30 min	OA/mPEG-PCC-VE NPs P_app_ Colon: 14 × 10^3^ cm/min P_app_ Ileum: 4 × 10^3^ cm/min P_app_ jejunum: 6 × 10^3^ cm/min P_app_ duodenum: 10 × 10^3^ cm/min	1.5-fold	[153]
	PLGA, polyphenols	OA-PLGA (74.5 ± 3.5) CH-OA-PLGA (71 ± 2.5) OA-B-PLGA (81.1 ± 2.1) CH-OA-B-PLGA (78.6 ± 1.3)	-	PLGA-NP (149.6 ± 3.4) OA-PLGA (184.5 ± 1.5) CH-OA-PLGA (326.8 ± 2.5) OA-B-PLGA (221.5 ± 1.9) CH-OA-B-PLGA (342.2 ± 3.7)	PLGA-NP (−23.1 ± 4.2) OA-PLGA (−23.4 ± 2.2) CH-OA-PLGA (29.8 ± 1.1) OA-B-PLGA (−27.2 ± 2.5) CH-OA-B-PLGA (34.2 ± 3.1)	PLGA-NP (0.30 ± 0.012) OA-PLGA (0.22 ± 0.032) CH-OA-PLGA (0.33 ± 0.043) OA-B-PLGA (0.21 ± 0.021) CH-OA-B-PLGA (0.22 ± 0.011)	OA (~90%) OA-PLGA (~75%) CH-OA-B-PLGA (~40%) CH-OA-PLGA (~50%) OA-B-PLGA (62%)	-	↑ antitumor effect on breast cancer cells in MDAMB 231 cell line	[154]
	PLGA	∼77%	-	˂225	−27 mV	0.1	75% in 40 h	Papp 3.36 ± 0.49 cm s^−1^	↑ anti-inflammatory effect	[133]
	OA and Paclitaxel (PTX)	-	-	OA NPs (247.7 ± 0.8) PTX-OA NPs (259.7 ± 3.4)	OA NPs (−8.8 ± 0.2 mV) PTX-OA NPs (−5.8 ± 0.6 mV)	OA NPs (0.035 ± 0.020) PTX-OA NPs (0.020 ± 0.008)	-	-	Synergistically ↑ antitumor activity to 69% from 15%	[155]
	OA loaded in liquid crystalline nanoparticle (LCNP)-based gel	68.31 ± 2.86% to 73.18 ± 3.21%	12.31 ± 0.41% to 14.12 ± 0.32%	129 ± 12.11 to 272 ± 21.83	− 18.3 to − 21.2	0.218 to 0.436	84.93 to 87.89% at 12 h	Pe (0.98 cm/h to 30.96 cm/h)	effective in a rodent carrageenan-induced hind paw inflammatory model	[156]

Abbreviations: OA: oleanolic acid, TPGS: vitamin E-D-α-tocopheryl polyethylene glycol succinate, ALB: albumin, CTX: cetuximab, UA: ursolic acid, PLGA: chitosan-coated Poly (lactide-co-glycolide), ALPs: *Actium lappa* polysaccharides.

**Figure 8:**
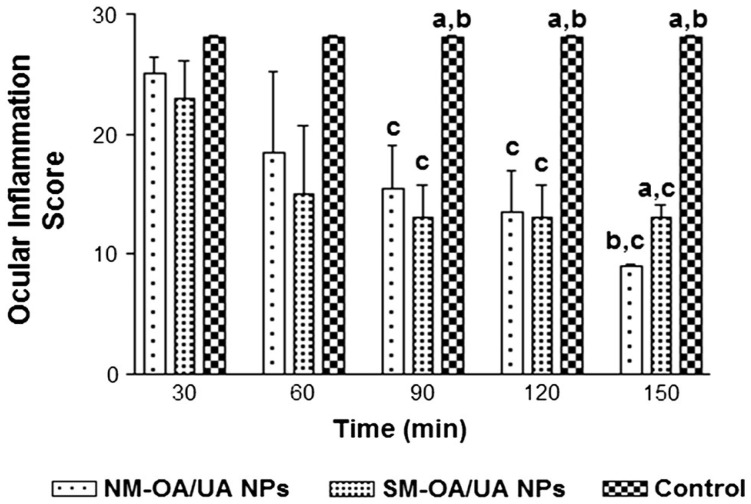
Anti-inflammatory efficacy of NPs after sodium arachidonate solution (SAS) induced inflammation in the rabbit eye. Statistically significant differences, *p* < 0.05, regarding ^a^ NM-OA/UA NPs, ^b^ SM-OA/UA NPs, and ^c^ Control (SAS). Reproduced with permission from [133], Elsevier, 2015.

In another inquiry, polyphenolics were extracted from *P. longum*, *P. nigrum*, and *Z. officinale*, then combined with OA in a 70:30 methanol:water ratio. The mixture was evaporated and lyophilized to yield the powdered polyherbal formulation OA-B (6:1:1:1 OA:*P. longum*:*P. nigrum*:*Z. officinale*). The OA-B (polyphenolic bio-enhancers) and OA were co-delivered in chitosan-coated poly (lactide-co-glycolide) (PLGA) NP for enhanced antitumor effects while preserving female fertility. The optimized oleanolic-bio-enhancer nanoformulation (CH-OA-B-PLGA) exhibited significant efficacy, inducing apoptosis in cancer cells and indicating enhanced antitumor potency in mice without compromising safety or fertility. This breakthrough suggests a promising approach for safe chemotherapy in hormone-independent breast cancer therapy, addressing toxicity concerns [154]. In a different investigation, polymeric nanoparticles using PLGA loaded with natural and synthetic mixtures (NM and SM) of OA and UA were designed for ophthalmic use. NM comprises naturally occurring forms of OA and UA obtained from plant sources, while SM denotes a mixture of synthetically produced OA and UA, as the major drawback of those natural compounds is the limited amount present in plants. The optimized formulations exhibited favorable properties, including small size (<225 nm), uniform distribution, negative surface charge (around −27 mV), and high entrapment efficiency (around 77%). Release and corneal permeation studies indicated faster release for NM, with higher drug retention in corneal tissue. Both formulations showed no irritation or toxicity in in vitro and in vivo tests, and demonstrated effective in vivo anti-inflammatory efficacy, with NM-OA/UA NPs being the most potent (Figure 8) [133].

In another investigation, OA was used to produce NPs capable of efficiently crossing the blood–brain barrier for the treatment of breast cancer brain metastases. Based on these observations, the scientists devised a synergistic combination chemotherapy approach by incorporating paclitaxel (PTX) into OA-NP. The resulting PTX-OA-NP demonstrated significant efficacy in inhibiting both primary breast cancer and breast cancer brain metastases in mouse xenografts [155].

A cubic liquid crystalline NP (LCNP)-based gel was developed as a potential topical delivery system for OA. Optimization using rheological, drug release kinetic, and ex vivo permeation studies yielded promising results. The optimized LCNP formulation demonstrated sustained release (85.49 ± 0.21%) over 12 h in vitro, and ex vivo permeation studies indicated its superiority to a standard gel. The Peppas equation suggested non-Fickian diffusion, implying multiple controlled release processes. The OA-loaded LCNP gel exhibited efficacy in a rodent inflammation model, providing sustained relief after a single application [156]. Efforts were made to enhance the solubility of OA and UA also using polyurethane nanostructures (PU) synthesized through interfacial polycondensation and spontaneous emulsification. Despite moderate chemo-preventive activity against induced tumors, encapsulating UA and OA in PU did not increase their effectiveness. Consequently, PU was deemed unsuitable as a formulation for UA and OA [2].

Similarly, a study investigated a combined chemotherapeutic response by pairing doxorubicin with human serum albumin-oleanolic acid NP (Dox@HSA-OA NP). In vitro studies demonstrated higher cellular association, lower IC50, increased apoptosis, and G2/M phase cell cycle arrest with Dox@HSA-OA NP compared to free doxorubicin. Spheroid studies further highlighted the efficacy of Dox@HSA-OA NP in inducing cell death. In vivo experiments with B16F10 tumor-bearing mice showed superior outcomes, including tumor regression, DNA damage, oxidative stress, and apoptosis induction, suggesting that Dox@HSA-OA NP could be a potent strategy for solid tumor treatment compared to doxorubicin alone [157]. Likewise, a pH-sensitive calcium carbonate NP was developed to co-deliver cisplatin (CDDP) and OA, aiming to enhance tumor efficacy while reducing nephrotoxicity. This approach combines the potent anticancer properties of CDDP with the nephroprotective effects of OA, offering a promising strategy for a safer and more effective combination chemotherapy against cancer. Additionally, the system enables targeted delivery to tumor sites, enhancing therapeutic outcomes while minimizing off-target effects. The microemulsion method produced lipid-coated cisplatin/oleanolic acid calcium carbonate nanoparticles (CDDP/OA-LCC NPs). The NPs exhibited an average diameter of 217 ± 20 nm, with a zeta potential of −23.7 ± 2 and a PdI of 0.187, indicating uniform dispersion. Drug loading percentages were determined to be 76 ± 5% for CDDP and 50 ± 7% for OA (*w*/*w*). In vitro assays validated their synergistic apoptotic effects on HepG2 cells, and this synergy translated to enhanced antitumor efficacy in a tumor-bearing mice model compared to free drugs. Pharmacokinetic studies showed a prolonged circulation time, and in vivo imaging indicated tumor-specific concentrations. OA markedly alleviated CDDP-induced nephrotoxicity. This pH-sensitive, dual-loaded NP offers a potential avenue for achieving a more effective and safer approach to combination chemotherapy [158].

In a follow-up study, the cancer cell membrane (CM)-decorated calcium carbonate (CC) hybrid NPs (HN) for the co-delivery of cisplatin (CDDP) and OA were developed. The HN/CDDP/OA system exhibited a core-shell structure, good dispersion, and a size of around 100 nm. It demonstrated high stability, biocompatibility, and pH-responsive drug release. Additionally, CM modification improved tumor-targeting capabilities compared to bare CC nanoparticles. In tests on gastric cancer (MGC-803 cell line), HN/CDDP/OA outperformed single-drug systems, inducing enhanced apoptosis and reversing multidrug resistance (MDR) in cancer cells.

Heparin sodium (HS)-loaded polylactic-co-glycolic acid-D-α-tocopheryl polyethylene glycol 1000 succinate (PLGA-TPGS) NPs (HPTNs) for sustained delivery were combined with OA-loaded PLGA-TPGS nanoparticles (OPTNs) to produce a synergistic therapy system for liver cancer treatment. Using fluorescent probes, efficient internalization into cells and enhanced liver targeting were observed. The combination of HPTNs and OPTNs exhibited effective cell inhibition and a remarkable anticancer effect, inducing apoptosis in HepG2 cells. In vivo studies showed a 67.61% suppression of tumor growth. The combination therapy system involving OPTNs and HPTNs presents a promising approach for hepatoma therapy [159].

### 5.5. Solid Dispersions

**Figure 9:**
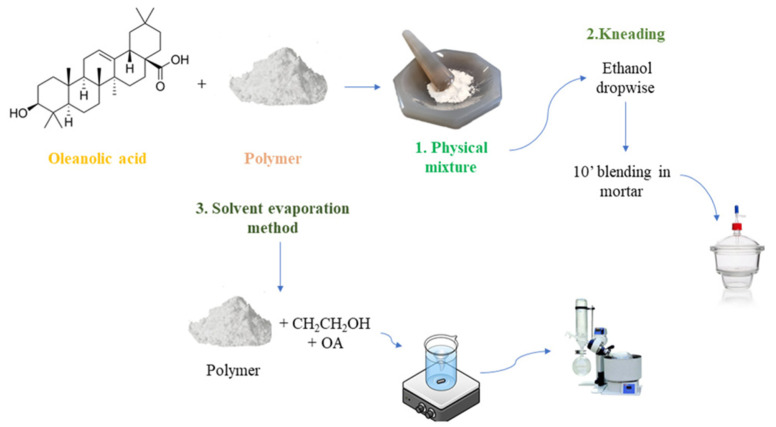
Schematic representation of preparation of the solid dispersions using the physical mixture, kneading, and the solvent evaporation method. Reproduced with permission from [164], MDPI, 2022.

**Figure 10:**
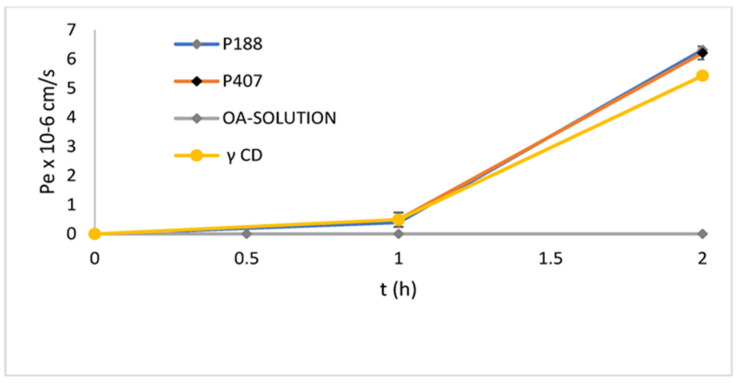
Effective permeability (Pe) of OA, SEM OA-P188 1:2, SEM OA-P407 1:2, and SEM OA-γ-CD1:2. SEM: solvent evaporation method, CD: cyclodextrin, P407: Poloxamer 407, P188: Poloxamer 188, OA: oleanolic acid. Reproduced with permission from [164], MDPI, 2022.

Solid dispersion (SD) involves dispersing one or more active ingredients in a carrier or matrix in a solid state, serving as an effective approach to enhance the dissolution of poorly water-soluble drugs and consequently improve their bioavailability [160]. SD technology is preferred for formulating BCS class-II/IV APIs, with a wide range of polymeric carriers available for formulation scientists [161]. Enhancing bioavailability through a SD system primarily involves enhancing the dissolution rate. This is achieved by improving the wetting behavior of hydrophobic drugs and facilitating deagglomeration and micellization with hydrophilic polymers (Table 4) [162]. A study was conducted to improve OA dissolution using SDs comprising the drug, a polymeric carrier, and a surfactant. Binary SDs (OA and PVP) were compared with ternary SDs, where polysorbate 80, a nonionic surfactant, was added to the binary form. Both binary and ternary SDs enhanced OA dissolution, with the ternary form exhibiting faster dissolution than the binary form. Polysorbate 80 played a central positive role in the dissolution of the SD [163]. Similarly, a SD was developed to address limitations due to the poor solubility of OA. Solubility studies assessed various hydrophilic polymers, drug-to-polymer ratios, and preparation methods, as shown in Figure 9, revealing Poloxamer 188, Poloxamer 407, and γ-CD as the most effective in increasing OA’s solubility. The preparation methods kneading and solvent evaporation and the drug-to-polymer weight ratio of 1:2 resulted as the best to improve the OA solubility up 190 μg/mL. In vitro dissolution studies for P407, P188, and γ-CD SDs showed the solvent evaporation method as the most effective in enhancing dissolution compared to the OA solution. An in vitro parallel artificial membrane permeability assay (PAMPA) demonstrated improved passive permeation, as demonstrated in Figure 10, indicating that the amorphization of OA by SD preparation could be a promising method for enhancing its oral absorption, applicable on an industrial scale [164].

**Table 4:** OA-loading solid dispersions and complexes: characteristics and performance evaluation. ↑: increase.

DDS	Components	In Vitro Release	Solubility	Permeability (Pe cm/s)	Activity and/or Bioavailability	References
SD	OA, PVP SD OA, PVP, Polysorbate 80 SD	70% 90%	-	-	-	[163]
	OA:Poloxamer 188 1:2 OA:Poloxamer 407 1:2 OA:γ-CD 1:2	61% 68% 50%	190 ± 42 μg/mL 170 ± 28 μg/mL 221 ± 17 μg/mL	OA-P407 6.2 ± 0.22 × 10^−5^ OA-P188 6.3 ± 0.53 × 10^−5^ OA-γ-CD 5.43 ± 0.12 × 10^−5^ at 2 h	-	[165]
	Formula F (OA:PVP:SC) 1:1:2 Formula G (OA-Na:PVP:SC) (1:1:2)	OA-Na (9.5%) Formula F (59%) Formula G (85%) at 2 h	-	OA 0.138 ± 0.023 0.06% (*w*/*v*) SC* 0.381 ± 0.053 at 2 h	Formula F (31,067.44 ± 17,840.92) Formula G (32,657.41 ± 11,832.92) (μg min/mL)	[165]
	OA-PVP Physical mixture (PM)-PVP	90% 45% at 2 h	-	-	AUC_0–∞_ (ng h/mL) OA-PVP: 2039.5 ± 483.4) PM-PVP: 696.8 ± 151.6 commercial tablet: 875.08 ± 292.1	[166]
	OA:silica 1:7	86.4%	-	-	AUC_0–24h_ OA-silica: 228.51 ± 20.35(μg min/mL) 1.9-fold increase	[167]
Phospholipid complexes	OA-phospholipid complex 8:5	99.47% in 360 min	300-fold	-	AUC_0–∞_ 2.16-fold	[168]
	OA, phospholipid complex and hydroxyapatite (OPCH)	90% in 30 min	water 15.3-fold n-octanol 3.19-fold	OPCH increased 1.6–2.6-fold compared to OA. OPCH in the presence of ketoconazole 1.2–2.4-fold compared to OPCH.	AUC_0–t_ (ng h/mL) OPCH: 360.6 ± 19.13 OPCH with ketoconazole: 707.7 ± 30.21	[19]
	OA, Phosphatidylcholine (PC), fumed silica	0.1% SDS aqueous solution: OA 20%, OA-PC 10%, OA-PC/silica ˃40% 0.3% SDS aqueous solution: OA ˃50%, OA-PC ˃20%, OA-PC/silica 80% 0.5% SDS aqueous solution: OA ˃60%, OA-PC ˃30%, OA-PC/silica ˃80%	OA-PC: 0.12 (30 °C), 0.14 (40 °C), 0.17 (60 °C), 0.15 (80 °C)	-	-	[1]
Other complexes	OA and methyl-β-CD	-	8.2	-	↑ in vitro anticancer activity in HepG2, HT29, and HCT116 cell lines	[169]
	2-hydroxypropyl-β-CD and 2-hydroxypropyl-γ-CD	-	-	-	↑ anti-proliferative A375 (human), B16 4A5 (murine), and SK-Mel 2 (human) cell lines	[170]
	hydroxypropyl-β-CD and OA and UA	-	OA (900 times) UA (200 times)	-	-	[171]
	amino-appended β-CDs	-	6.8 × 10^5^ to 2.1 × 10^6^ fold	-	↑ in vitro anticancer activities in HepG2, HT29, and HCT116 human cancer cell lines	[172]

Abbreviations: OA: oleanolic acid, SD: solid dispersions, CD: cyclodextrin, SC: sodium caprate, UA: ursolic acid, PVP: polyvinylpyrrolidone. Note: SC*: 0.06% (*w*/*v*) of SC increased the permeation of OA through the Caco-2 cell monolayer in 2 h.

In a similar fashion, spray-freeze-drying (SFD) of OA with PVP-40 and sodium caprate (SC) produced amorphous SD systems. These formulations showed superior dissolution and more consistent absorption than commercial OA tablets. SC increased in vivo absorption rates without affecting the absorption extent. However, both SFD-processed OA and commercial tablets exhibited significant inter-animal variability in bioavailability, common for BCS Class IV compounds. Introducing SC and using the sodium salt of OA (OA-Na) reduced the absorption variability, emphasizing the importance of enhancing both the dissolution rate and intestinal permeability for BCS Class IV compounds [165]. In another study, hot-melt extrusion was employed to make amorphous SD of OA. The resulting OA SD with polyvinylpyrrolidone vinyl acetate copolymer (OA-PVP) exhibited significantly enhanced dissolution rates compared to a physical mixture (PM-PVP) and a commercial tablet in a medium containing 0.3% SDS. Moreover, OA-PVP exhibited higher AUC and C_max_ values than both PM-PVP and the commercial tablet, indicating improved bioavailability. The successful outcome was attributed to the amorphous state of OA in PVP, as well as effective dispersion achieved through thermal melting and shearing [166]. Another study aimed to enhance the dissolution rate and oral bioavailability of OA in beagle dogs by adsorbing OA onto fumed silica using supercritical carbon dioxide. The optimal OA-silica SD demonstrated a significantly improved dissolution rate compared to commercial tablets and physical mixtures in dissolution tests. Bioavailability results in beagle dogs indicated that OA-silica solid dispersions had higher AUC and C_max_ than commercial tablets (*p* < 0.05), resulting in a 1.9-fold increase in bioavailability for OA absorption from SD [167].

### 5.6. Phospholipid Complexes

Recently, the phospholipid-complex technique has been applied for its solubilizing capacity or its potentiating ability to pass through the biological membranes and its protection of the active herbal compounds from degradation. The phospholipid complex technique can serve as a powerful drug delivery system to increase the therapeutic index of encapsulated herbal active ingredients. Different sources contribute to the diversity of phospholipids, such as soybean, egg, or synthetic phosphatidylcholine, including hydrogenated forms. These variations make phospholipids a versatile option for drug delivery systems, enhancing oral bioavailability and improving drug solubility and permeability [173].

Thus, formulation scientists have tried to enhance the solubility and bioavailability of OA, producing the OA-phospholipid complex (OA-PLC; Table 4). Solubility in water rose by 300 times and in n-octanol by 1.2 times. Rats treated with OA-PLC showed a C_max_ of 1.18 µg/mL, versus 0.47 µg/mL for OA. AUC_0–24h_ and AUC_0–∞_ values in the OA-PLC group were particularly higher, 2.06 and 2.16 times, than those in the OA group, indicating OA-PLC’s superior solubility and enhanced bioavailability in rats [168]. In another study, a solidified phospholipid complex (OPCH) was prepared using OA-phospholipid complex (OPC) and hydroxyapatite (HA) through solvent evaporation. OPCH, co-administered with ketoconazole (KCZ), enhanced OA’s bioavailability by increasing water and n-octanol solubility, with a 15.3-fold and 3.19-fold increase, respectively. In vitro dissolution and intestinal perfusion studies revealed that OPCH’s cumulative dissolution rate was 2.23-fold and 4.57-fold higher than OA and OPC at 2 h. KCZ further improved absorption by inhibiting OA metabolism by CYP3A. Pharmacokinetic analysis in rats demonstrated a marked increase in C_max_ and AUC_0–24h_, indicating that OPCH formulation and KCZ co-administration significantly enhanced OA’s bioavailability by improving solubility and permeability, and inhibiting metabolism [19]. The dissolution rate of the formulated solidified powder using fumed silica as a carrier exceeded that of both pure OA and the OA-PC. The OA-PC complex exhibited a lower dissolution rate than crude OA, which might be due to the viscous nature of the phospholipid complex. Regarding solubility, OA-PC solubility increased with temperatures from 30 to 60 °C but decreased at 80 °C, possibly due to phospholipid oxidation [1].

### 5.7. Other Complexes

A new oral nanocomplex combining deoxycholic acid-grafted low-molecular-weight chitosan and OA (LW-CS-DA/OA) effectively reversed CCl4-induced acute liver damage in mice. These nanocomplexes exhibited high drug loading (26.74%) and encapsulation efficiency (78.50%), with a size of 239.50 ± 2.40 nm and a PdI 0.117 ± 0.0085. LW-CS-DA/OA reached a maximum plasma concentration of 23.97 μg/mL at 8 h, approximately 5 times higher than the OA group (1.5 h). These nanocomplexes showed better liver protection by enhancing the antioxidant capacity and regulating inflammation pathways. This innovative approach offers a promising strategy for chemical-induced liver injuries [174].

Another study aimed to enhance OA and UA properties by forming inclusion complexes with hydrophilic cyclodextrins. After virtual screening, 2-hydroxypropyl-β-cyclodextrin (HP-β-CD) and 2-hydroxypropyl-γ-cyclodextrin (HP-γ-CD) were selected. In vitro tests on melanoma cell lines showed increased anti-proliferative activity for the complexes compared to pure compounds. The UA-HP-γ-CD complex exhibited the highest activity. This approach of entrapping compounds in hydrophilic cyclodextrins proved effective in improving their anti-proliferative activities [170]. Another study explored ethanol’s impact on forming complexes of HP-β-CD with OA and UA. Phase solubility studies, including ethanol, assessed HP-β-CD’s solubilizing effect on OA and UA with a predictive mathematical model. Solid complexes, achieved by evaporating filtrates, significantly increased OA and UA solubility. Ethanol (0.5%, *v*/*v*) facilitated OA-HP-β-CD complex formation but hindered UA-HP-β-CD complex formation. Optimal solubility enhancement was achieved by adding ethanol [171].

An investigation into the solid inclusion complexes of OA with a series of amino-appended β-cyclodextrins (ACDs) was carried out using the suspension method. ACDs are β-cyclodextrins modified with amino side chains of varying lengths at the primary face. These modifications aim to improve the water solubility of β-cyclodextrins. The inclusion complexation with ACDs substantially improved the water solubility of OA. Furthermore, the in vitro anticancer activities of OA against the human cancer cell lines HepG2, HT29, and HCT116 showed significant enhancement upon the formation of inclusion complexes [172]. In a follow-up investigation, a solid inclusion complex of OA with methyl-β-cyclodextrin was synthesized. The inclusion complexation significantly enhanced the water solubility of OA to 8.2 mg/mL. The inclusion complex demonstrated significant enhancement of the in vitro cytotoxicity compared to native OA, with IC50 values of 8.89, 7.89, and 5.77 μM on the human cancer cell lines HepG2, HT29, and HCT116, respectively, as determined by the MTT assay [169].

### 5.8. Other Formulations

Expanding upon the comprehensive review of OA drug delivery techniques, this section explores additional strategies for elucidating further advancements in the field. In an investigation, OA was incorporated into poly(lactic-co-glycolic) acid fiber membranes. The resulting fiber membranes, developed with varying OA concentrations, exhibited high yields and average fiber diameters ranging from 541 to 630 nm with approximately 28% dissolution. These membranes, composed of long and continuous fibers with stable, rough surfaces in aqueous media, demonstrated structural and thermal stability. High drug loading efficiency (>80%) and non-toxicity to fibroblast cells were observed, suggesting these membranes as a promising vehicle for drug delivery applications due to their stability and biocompatibility [175].

Another investigation used a salt of OA and choline as the natural product hydrogelator to make low-molecular-weight supramolecular hydrogels (LMWSHs) by heating. Unlike typical sol–gel transition, the OA-based LMWSH exhibited a unique property, transitioning from a sol to a gel state upon heating, accompanied by phase separation with nonreversible transparent–turbid transitions. These LMWSHs demonstrated stability, injectability, and potential as drug delivery vehicles for sustained release [176].

In the milieu of OA for osteoarthritis treatment, a scaffold has been developed to counteract the detrimental effects of inflammatory cytokines on chondrocytes and enhance the repair of cartilage defects using tissue engineering. This involved grafting a berberine-oleanolic acid complex salt (BOA) onto hyaluronic acid (HA) to make water-soluble BOA-g-HA, which was then mixed with 30 mg/mL of silk fibroin (SF) to form four solutions. These solutions were lyophilized to produce composite scaffolds, denoted as BOA-g-HA/SF-1, BOA-g-HA/SF-2, BOA-g-HA/SF-3, and BOA-g-HA/SF-4, containing 0.75, 1.5, 2.25, and 3.0 mg/mL of BOA-g-HA, respectively. Particularly, the BOA-g-HA/SF-3 scaffold demonstrated the highest efficacy in maintaining the chondrocytic phenotype under IL-1β-induced stress, both in vitro and in vivo, showing significant regeneration of cartilage tissue [177]. Metal-organic frameworks (MOFs) were explored as drug carriers for OA delivery. A hollow MOF-5 structure was synthesized, increasing the drug loading capacity (42.43%) and achieving sustained-release effects (81.2%, 30 h). Evaluated on the SK-OV-3 cell line, it demonstrated improved treatment efficiency for OA. This highlights the potential of hollow MOFs as efficient drug carriers [178]. Another study aimed to make oleanolic acid nanofibers (OAnf) and test their effects on keratinocytes exposed to particulate matter (PM). OAnf has reduced PM-induced oxidative stress and inflammation in keratinocytes more effectively than OA alone. The formulation parameters of OAnf included a particle size of 302.37 ± 11.91 nm and a PdI of 0.32 ± 0.02. Drug loading was 98.19 ± 4.82%, and the encapsulation efficiency was greater than 99%. OAnf significantly improved water solubility (998.7 ± 58.32 µg/mL) and skin penetration (45.27 µg/cm^2^) at 4 h compared to OA alone [179].

## 6. Expert Opinion

Innovative formulations, including a variety of lipid-based delivery systems, such as SLN and NLC, micro- and nano-emulsions, liposomes, micelles, nanosuspensions, nanoparticles, solid dispersions, and phospholipid complexes, are essential in drug delivery applications. Micro- and nano-carriers are an important field of research in bioactive constituents, which include natural products and herbal extracts. The use of these technologies has already had a significant impact on many areas of medicine by allowing appropriate therapeutic treatments for certain essential drugs.

These carriers offer numerous advantages, particularly, enhancing the solubility and bioavailability of hydrophobic drugs, improving the stability of drug compounds, and the potential for controlled release and targeted delivery. Furthermore, they often demonstrate compatibility with diverse administration routes [180,181,182,183].

In comparing the formulations reported in this review, it can generally be stated that lipid-based drug delivery systems and NP are the nanocarriers more efficient for OA delivery. In particular, ME and NE are able to load high doses of a drug, control the rate and extent of drug absorption, increase solubility and bioavailability, enhance chemical stability, and overcome multidrug-resistance phenomena [184].

Polymeric NP can address biocompatibility and biodegradability and their resulting safety issues, especially after long-term administration. In the literature, different examples confirmed their effectiveness for OA delivery, increasing solubility, bioavailability, pharmacological efficacy, and targeting action.

Similarly, nanosuspensions address distinct challenges in drug delivery by maintaining the OA in a crystalline state while facilitating higher drug loading during formulation development. Additionally, they offer benefits such as improved stability, sustained drug release, enhanced efficacy through targeted tissue delivery, minimized first-pass metabolism, and enhanced deposition in the deep lung [144]. Liposomes, for instance, offer high biocompatibility but suffer from limited drug loading capacity and high production costs [185]. Solid dispersions and phospholipid complexes enhance the OA dissolution profile, resulting in increased bioavailability and stability due to their solid form. The phospholipid-complex technique has been applied for its solubilizing capacity or its potentiating ability to pass through the biological membranes.

For all these reasons, encapsulation methods remain indispensable in pharmaceutical development, offering customized solutions to improve drug delivery efficiency and therapeutic outcomes. Each technique presents specific advantages but also some disadvantages, including limited drug loading capacity, difficult scalability, low storage stability, and potential toxicity or irritation due to the presence of surfactants and co-solvents [180,182].

Exploring the numerous pharmacological effects, molecular mechanisms, and diverse delivery modalities for OA, it is important to acknowledge potential limitations in the existing approaches. Despite significant advancements in drug delivery systems for OA, several challenges persist. These may include issues related to bioavailability, stability, and targeting efficiency. Moreover, the variability in experimental conditions and methodologies across studies can hinder direct comparisons and the generalizability of findings. Expert opinions suggest that further research is needed to address these limitations and propel the field forward.

Additionally, it is worth noting that, while extensive preclinical studies have been performed, limited or no clinical studies have yet been conducted on the efficacy and safety of OA formulations in human subjects. This critical gap in knowledge emphasizes the need for comprehensive studies to advance our understanding of OA’s potential applications and efficacy in both preclinical and clinical settings.

## 7. Conclusions

The increasing consideration of OA as a potent triterpenoid, well known for its diverse health benefits, emphasizes the significance of exploring its application in pharmaceutical research. OA’s pharmacological effects highlight its therapeutic potential. However, realizing this potential is based on overcoming challenges in drug delivery, such as poor solubility and limited bioavailability. This review emphasized the necessity for innovative DDS and discussed various approaches aimed at enhancing OA’s biopharmaceutical features. The ongoing advancements in OA formulations aim to improve solubility, permeability, and overall efficacy across diverse therapeutic landscapes.

## Data Availability

Data sharing is not applicable to this article.
